# Noncoding RNAs

**DOI:** 10.1155/2012/629249

**Published:** 2012-12-29

**Authors:** Anandwardhan A. Hardikar, Michael D. Walker, Francis Lynn

**Affiliations:** ^1^NHMRC Clinical Trials Centre, University of Sydney, Level 6, Medical Foundation Building, 92-94 Parramatta Road, Camperdown, NSW 2050, Australia; ^2^Department of Biological Chemistry, Weizmann Institute of Science, 76100, Rehovot Ullmann Building, 3rd Floor, Rooms 341 and 336, Israel; ^3^Child and Family Research Institute, University of British Columbia, Canada

Recent years have witnessed many remarkable discoveries in the life sciences that have led to major paradigm shifts. An example is the emerging appreciation of the critical role of noncoding RNAs in a broad range of physiological and pathological processes. One such class of noncoding RNAs, the microRNAs (miRNAs), controls intricate networks of gene expression via post-transcriptional mechanisms. Originally described in animals by V. Ambros and G. Ruvkin in 1993, microRNAs are now recognized to impact human development and health in many ways. Another distinct and rapidly growing class of noncoding RNA, the long ncRNAs (lncRNAs), is also implicated in gene regulation and disease.

Across the phylogenetic kingdoms, bacteria and other prokaryotes have proportionally the least amount of noncoding DNA. Interestingly the total number of protein-coding genes does not consistently increase with increasing complexity of organisms; for example, insects have just twice as many protein-coding genes (~13,500) than yeast (~6,000). Increasing cellularity also does not seem to affect the number of protein-coding genes in the genome. For example *C*. *elegans*, a worm that is well studied in developmental biology, has around 19,000 protein-coding genes that govern normal development and function of its 959 cells; whereas a similar number of protein coding genes (~25,000) in humans drives development and function of over 10 trillion cells. One major difference between the worm and human genomes is the relative proportion of noncoding DNA, which is seen to increase with complexity of body plan and cellular processes. In recent years it has been demonstrated that much of this “junk DNA” is transcribed as noncoding RNAs (including microRNAs and lncRNAs). Together, these noncoding RNAs have been demonstrated to influence the expression of around 30% of protein-coding genes and have been demonstrated to play important homeostatic roles that act to normalize gene expression and maintain cellular phenotypes during development and disease.

In this special issue focusing on noncoding RNAs, we bring together a collection of articles from various areas of diabetes and islet cell biology. T. Avnit-Sagi et al. and S. Kredo-Russo et al. discuss the regulation of two important pancreas-specific microRNAs, miR-7 and miR-375. Another article by A. D. Mandelbaum et al. describes how miRNAs can affect islet architecture. Research carried out in the past few years from several laboratories has clearly demonstrated that miRNAs and other noncoding RNAs are essential for development and differentiation of pancreatic progenitor cells as well as for regulation of glucose metabolism. An interesting application came through cancer research wherein specific noncoding RNAs were shown to be present at high abundance in the circulation. The possibility that microRNAs can be released in circulation as free microRNAs from dead/dying cells or specifically through exosomes has now been validated. An important area of research that stems from such observations is the possibility to utilize small noncoding RNAs as biomarkers for disease progression. The studies presented by L. B. Nielsen et al. and C. J. Taylor et al. in this issue are important in understanding and establishing microRNA-based biomarkers that can be used for prediction of diabetes and/or complications. Some of the above studies are reviewed in this issue by M. D. Williams et al. With the emergence of novel technologies, there will likely be many more discoveries linking noncoding RNAs with the development of diabetes. Given the pervasive impact of noncoding RNAs on cellular function, these articles provide a flavour of the wide range of biologies that are controlled by these previously unappreciated RNA molecules.

*Anandwardhan A. Hardikar* *Anandwardhan A. Hardikar* *Michael D. Walker* *Michael D. Walker* *Francis Lynn* *Francis Lynn*

